# Fine Tuning Cell Migration by a Disintegrin and Metalloproteinases

**DOI:** 10.1155/2017/9621724

**Published:** 2017-02-05

**Authors:** D. Dreymueller, K. Theodorou, M. Donners, A. Ludwig

**Affiliations:** ^1^Institute of Pharmacology and Toxicology, Uniklinik RWTH Aachen, Aachen, Germany; ^2^Department of Pathology, Maastricht University, LK Maastricht, Netherlands

## Abstract

Cell migration is an instrumental process involved in organ development, tissue homeostasis, and various physiological processes and also in numerous pathologies. Both basic cell migration and migration towards chemotactic stimulus consist of changes in cell polarity and cytoskeletal rearrangement, cell detachment from, invasion through, and reattachment to their neighboring cells, and numerous interactions with the extracellular matrix. The different steps of immune cell, tissue cell, or cancer cell migration are tightly coordinated in time and place by growth factors, cytokines/chemokines, adhesion molecules, and receptors for these ligands. This review describes how a disintegrin and metalloproteinases interfere with several steps of cell migration, either by proteolytic cleavage of such molecules or by functions independent of proteolytic activity.

## 1. Principles of Cell Migration

Cell migration is a critical step in the homeostatic and inflammatory trafficking of immune cells, the migration of cells during embryogenesis, in regenerative processes such as wound healing, in tissue homeostasis, and also in the development of diseases such as cancer [1]. Dysregulation in migration can result in severe peri- or postnatal defects such as the neural tube defect [2], heart abnormalities, and defective lymphopoieses [3, 4]. Further, in the adult organism, several pathologies are linked to alterations in migration, including inflammatory disorders such as rheumatoid arthritis and multiple sclerosis, vascular diseases [5], in which immune cells promote the inflammatory process [6], delay of wound closure, and tumor metastasis formation [7]. The list of migrating cell types is long differing in their form and speed of migration including immune cells, epithelial cells, endothelial cells, smooth muscle cells, pericytes, and neural cells. The exact mechanisms of cell migration can differ especially between rapidly migrating leukocytes and tissue cells. However, the involved surface molecules, the signal transduction pathways, and the underlying molecular machinery show a considerable degree of overlap in all motile cells.

On the migrating cell itself, a well-orchestrated sequence of single steps can be observed such as polarity changes, protrusion and retraction, and loose and firm adhesion to other cells or the extracellular matrix (ECM). Leukocytes and also cancer cells are capable of transmigrating through the tissue layers including endothelium or epithelium [7]. This also involves interaction with these tissue layers, which often regulate adhesion and junction molecules, thereby increasing permeability of the cell layer [8] as well as transmigration of the migrating cells. The polarizing and initiating stimulus can be of various nature: chemotactic (i.e., chemoattractants and morphogens) [1]; haptotactic (i.e., varying substrate concentrations in wound healing, angiogenesis, and metastasis) [9]; mechanotactic (i.e., loss of cell-cell contacts in wound healing or metastasis) [10]; durotactic (i.e., varying rigidity) [11]. Polarization is accompanied by the extension of generally formed pseudopods towards the direction of migration, driven by the rearrangement of the actin cytoskeleton [12]. The different protrusions mediate the interaction with surrounding tissue cells and the ECM and the formation of adhesive complexes. The presence of nascent adhesions and focal complexes are markers of fast migrating cells, whereas focal adhesions as more mature structures are inversely correlated with cell motility [13]. The most important common components of adhesive complexes are integrins as adhesion receptors. Integrins are cell specifically expressed and activated upon specific stimulation, thereby mediating leukocyte adhesion and transmigration [14]. Podosomes are found in fast moving cells such as macrophages, sharing similar structures with invadopodia of metastatic tumor cells [15]. Both include the redirection of integrin receptors and adhesion molecules to the leading edge of the migrating cells, while invadopodia further concentrate proteolytic components that degrade the surrounding matrix to facilitate transmigration [16]. Often, tissue or cancer cell migration requires the acquisition of a migratory phenotype. These phenotypic changes can be brought about by cytokines, growth, or differentiation factors. For example, repair processes involving tissue cell migration and also cancer cell migration can be initiated within the tissue layers by transforming growth factor (TGF) *α* and heparin-binding epidermal growth factor (HB-EGF) [17–19].

One of the most studied migratory events is the recruitment of immune cells from the blood to a site of inflammation, for example, caused by wounding or infection. Proinflammatory signals are released and relayed to the vascular endothelium, which exposes new adhesion molecules and receptors (e.g., E-selectin and P-selectin, vascular adhesion molecule 1 (VCAM-1), intercellular adhesion molecule 1 (ICAM-1), CXCL16, and CX3CL1 [20–23]). Immune cells are slowed down in migration and loosely adhering to the endothelium, rolling along the endothelium via the interaction of selectins with glycoprotein ligands, adhere more tightly via activated integrins, crawl on the endothelium probing for an extravasation point, and at last transmigrate through the endothelium.

Thus, for effective migration of immune cells, tissue cells or cancer cells several migratory steps need to be tightly coordinated. This involves the regulation of cytokines, growth factors, chemokines, adhesion molecules, and receptors for these ligands. Notably, many of these molecules are expressed as membrane-bound form and are functionally modulated by limited proteolysis close to the plasma membrane, a process called shedding. In many cases, members of the family of a disintegrin and metalloproteinases (ADAMs) mediate these shedding events. By this activity, ADAMs can interfere with several steps of cell migration. In addition, some ADAM family members can also regulate adhesion processes independently of any proteolytic activity.

## 2. ADAM Proteases

**Figure 1:**
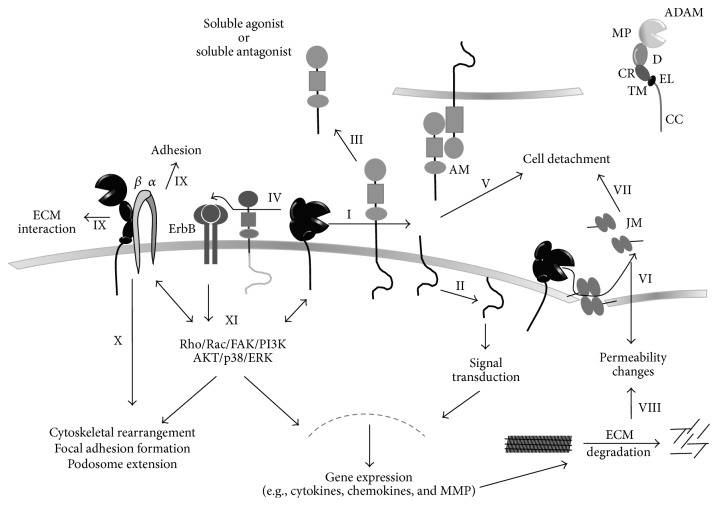
Catalytic and noncatalytic functions of ADAM proteases facilitating cell migration. ADAM proteases interfere with distinct steps of cell migration via a number different effector molecules. This involves proteolytic cleavage or nonproteolytic interactions of the proteases. ADAMs consist of a C-terminal cytoplasmic tail (CC) followed by a transmembrane domain (TM), an EGF-like domain (EL), a cysteine-rich domain (CR), and a disintegrin domain (D) and an extracellular metalloproteinase domain (MP) (ADAM). ADAM proteases cleave transmembrane surface molecules close to the plasma membrane, a process called shedding, requiring a zinc atom in the active site of the metalloproteinase domain (I). This results in the release of a soluble ectodomain and the production of a cell-associated fragment consisting of the transmembrane and cytoplasmic domain. The remaining fragment can undergo further regulated intramembrane proteolysis by *γ*-secretase (II) at last functioning as transcription factor (e.g., Notch). ADAM activity can be regulated by activation of a precursor protein (e.g., pro-MMP9). The released ectodomain may function either as agonist like the chemokine CXCL16 and the ErbB ligands HB-EGF and neuregulin or as antagonist like soluble JAM-A or RAGE (III). The release of ErbB ligands leads to transactivation of ErbB receptors in cis and trans (IV). The inactivation of ligands (i.e., adhesion molecules (AM)) can lead to the detachment of bound receptor complexes and attached cells (i.e., L-selectin, MAC-1) (V). The cleavage of junction molecules (JM, i.e., cadherins) results in changes of permeability (VI), facilitating the transmigration of inflammatory cells, the invasion of cancer cells, and the dissemination resulting in metastasis and angiogenic processes (VII). The permeability is further regulated by ECM degradation, by either direct action of ADAM proteases (i.e., ADAM9) or the regulation of MMPs (VIII). With their disintegrin-like domain, ADAMs can directly interact with adhesion molecules and ECM proteins (IX). The integrin (indicated by *α*/*β*) interaction/activation results in cytoskeletal rearrangement, focal adhesion formation, and podosome extension (X). Integrin, ADAM activation, and ErbB receptor transactivation result in different signaling pathways including the activation of Rho, Rac, FAK, PI3K, AKT, p38, and ERK, all further regulating ADAM and integrin activity as well as gene expression (XI). The exact mechanism of cell migration can differ between rapidly migrating leukocytes and tissue cells. However, the involved surface molecules, the signal transduction pathways, and the molecular machinery show a considerable degree of overlap for the action of ADAM proteases in inflammatory cell recruitment, angiogenesis, reepithelialization, cancer cell detachment and adhesion, and the intra- and extravasation during metastasis.

ADAM proteases belong to the class of metalloproteinases, which also comprises the matrix metalloproteinases (MMP) and a disintegrin and metalloproteinase with thrombospondin motif (ADAMTS). The ADAM family consists of 34 members, with 22 members described in humans [24, 25]. Most ADAMs are expressed as type I transmembrane surface proteins with a typical multidomain structure consisting of an N-terminal metalloproteinase domain, a disintegrin domain, a cysteine-rich domain, an EGF-like domain followed by a transmembrane domain, and a cytoplasmic tail (Figure 1). Most ADAMs are synthesized as proenzymes, in which the N-terminal and inhibiting prodomain is released by furin-mediated cleavage during the maturation process. The zinc-dependent metalloproteinase domain mediates the proteolytic activity, which is only present in ADAM8, ADAM9, ADAM10, ADAM12, ADAM15, ADAM17, ADAM19, ADAM20, ADAM21, ADAM28, ADAM30, and ADAM33 [26]. ADAM2, ADAM7, ADAM11, ADAM18, ADAM22, ADAM23, ADAM29, and ADAM32 lack the consensus sequence HExGHxxGxxHD for metalloproteinase activity. Their function remains largely unknown but may include functions as adhesion molecules rather than as proteases. The cysteine-rich domain and disintegrin domain of most ADAM proteases are involved in substrate recognition and interaction with integrins and the extracellular matrix. ADAM10 and ADAM17 do not carry the typical EGF-like domain. Their cysteine-rich domain is followed by a so-called membrane-proximal domain as well as a small stalk region [27–29]. The cytoplasmic tail is very diverse in length and may be involved in signaling between the intracellular and extracellular portion as well as in assembling of cytoplasmic adaptor molecules. Some ADAM proteases exist not only as transmembrane proteins but also as soluble forms resulting from splice variants. These soluble forms of ADAM9, ADAM12, and ADAM28 lack the transmembrane domain and the C-terminal cytoplasmic tail [26].

Proteolytic activity has been reported for ADAM8, ADAM9, ADAM10, ADAM12, ADAM15, ADAM17, ADAM28, and ADAM33 [30]. The interaction partners and substrates of ADAMs are generally transmembrane surface proteins. It is important to note that, with only one reported exception [29], shedding occurs in cis and not in trans, meaning that substrate and protease have to be expressed on the same cell. The regulated proteolysis occurs close to the cell surface, resulting in the release of a soluble ectodomain and the production of a cell-associated fragment consisting of the transmembrane and the cytoplasmic domain. After this shedding process, the remaining fragment can undergo further regulated intramembrane proteolysis (“RIPping”), which has been reported for Notch [31]. ADAM activity can convert surface molecules into soluble agonists. By this, ADAMs critically drive the activity of cytokines (e.g., TNF), chemoattractants (e.g., CX3CL1), or growth factors (e.g., EGF). Further, released ectodomains can act as antagonists and sequester soluble ligands (e.g., TNFR). Shedding of a receptor or adhesion molecule (e.g., L-selectin) can lead to reduced responsiveness (e.g., VEGFR2) or cell adhesion (e.g., RAGE, L-selectin). Moreover, proteolytic release of an adhesion molecule bound to its receptor can result in detachment of adherent cells. Finally, cytoplasmic fragments released by RIPping may function as transcription factors (e.g., Notch). Notably, ADAMs can directly interact with adhesion molecules and extracellular matrix proteins (e.g., collagen). This interaction involves the disintegrin domain and can promote cell-to-cell adhesion or strengthen barrier function of cell layers [32].

This broad activity requires a regulatory network to limit the proteases activity in space and time. It is not yet fully understood how the substrate selectivity of the single ADAM proteases is mediated, as the substrates do not seem to carry a distinct cleavage site [33, 34]. Specificity is possibly mediated by the substrate binding pocket [35], by exosites such as the cysteine-rich domain [29], the juxtamembrane region, and the transmembrane domain [27, 36–38]. Further, involved signaling pathways [39] as well as changes in the membrane structure [40, 41] may influence ADAM substrate specificity. Regulation of ADAM protease activity can occur on various levels. Acute and chronic stimulation and the tumor environment have been shown to enhance ADAM protease gene expression [32, 42, 43]. The increase of gene expression and enzyme synthesis are rather slow regulators of shedding activity. Rapid regulation of proteolytic activity occurs on the posttranscriptional level including the removal of the inhibitory prodomain [44], changes in membrane distribution [41, 45], release and transport to the cell surface [46, 47], multimerization on the cell surface [48], autocatalytic activation [49], interaction with adaptor molecules, and conformational changes [40, 50, 51]. Recently, pseudoproteases of the rhomboid family, the inactive rhomboid (iRHOM) 1 and 2, have been discovered as adapter molecules of ADAM17 in the endoplasmic reticulum mediating the transport of the protease to the Golgi apparatus and the cell surface [52, 53]. ADAM10 does not seem to interact with iRHOMs, but its surface expression strongly depends on tetraspanins of the TSPANC8 family [54, 55]. Interestingly, also ADAM17 is regulated by the tetraspanin CD9/TSPAN29 [56]. Since iRHOMs and tetraspanins are critical for ADAM activity on the cell surface, they very likely also interfere with ADAM-dependent mechanisms of cell migration. Furthermore, tissue inhibitors of metalloproteinases are well known to block ADAM proteases. TIMP-1 turned out to be a relevant inhibitor of ADAM10 [57], while TIMP-3 is more effective on ADAM17 [58]. Thus, regulation of TIMP1 or 3 also represents a means of controlling ADAM10- or ADAM17-mediated migratory processes. As discussed below, rapid posttranslational regulation of shedding events and long-term transcriptional induction of protease activity contribute to very basic migratory events involved in many different pathologies including inflammation, healing responses, and tumor metastasis.

## 3. ADAM Functions in Leukocyte Migration

As mentioned above, ADAM proteases may regulate cell migration either by their shedding activity or by the noncatalytic functions such as matrix and integrin interactions (Figure 1).

### 3.1. ADAM-Mediated Shedding in Leukocyte Migration

Leukocyte migration through tissue cells can depend not only on the proteolytic activity of ADAMs on the leukocytes themselves (see Table 1) but also on the ADAM protease activity on the tissue cells serving as substrate of cell migration (see Table 2).

In tissue cells transmigrated by leukocytes, ADAM10 and ADAM17 are the predominant proteases mediating shedding events that regulate leukocyte migration. Both endothelial and epithelial cells form a dense barrier to insure tissue integrity, which has to be opened transiently for transmigration. Upon stimulation with VEGF or thrombin, VE-cadherin is shed from the endothelial cell surface by ADAM10 and ADAM17 [59, 60]. The epithelial counterpart E-cadherin is solely shed by ADAM10 under physiological as well as pathological conditions [61]. Whereas shedding of VE-cadherin and E-cadherin has been shown to facilitate transmigration, the function of JAM-A is divergent. In its transmembrane form, JAM-A controls endothelial tight junction formation and contributes to leukocyte transendothelial migration [62, 63]. In contrast, soluble JAM-A, predominantly released by ADAM17, was shown to reduce inflammatory cell recruitment both in vitro and in vivo, avoiding excessive infiltration [62]. Endothelial cells express the transmembrane chemokines CXCL16 and CX3CL1, which are upregulated under inflammatory conditions and constitutively and inducibly shed by ADAM10 and ADAM17 [22, 64, 65]. The transmembrane forms act as adhesion molecules, regulating the interaction of T lymphocytes and monocytes with tissue cells [66, 67], whereas the soluble variants act as chemokines, mediating the recruitment of CXCR6 or CX3CR1 expressing cells to the site of inflammation [68–73]. In inflammatory diseases, CX3CL1 is released not only by the endothelium but also by monocytes, T lymphocytes, and NK cells as shown for rheumatoid arthritis and cardiovascular diseases [74] and further enhances the recruitment of inflammatory cells from the circulation.

Meprins A and B, receptor for advanced glycation end products (RAGE), and activated leukocyte cell adhesion molecule (ALCAM (CD166)) are additional substrates shared by leukocytes and tissue cells in leukocyte migration. The meprins are shed by ADAM10 [75, 76] releasing a soluble form and facilitating migration through the cleavage of ECM molecules [77]. Additionally, meprin B cleaves E-cadherin, enhancing the induction of epithelial permeability [78], which could be negatively regulated by ADAM10. Trauma or inflammatory stimulation leads to the upregulation of RAGE on epithelial and endothelial cells, facilitating the adhesion and subsequent diapedesis of leukocytes [79, 80]. The function of RAGE on leukocytes appears to be cell-type dependent. For macrophages, dendritic cells and T cells' influences only on activation, differentiation, and proliferation were reported. However, on neutrophils, RAGE was described as a chemotactic receptor interacting with high mobility box 1 protein (HMGB1) [81]. It is important to note that, in contrast to transmembrane RAGE, soluble RAGE released by ADAM10 functions as negative regulator of adhesion, blocking the activation of MAPK3 and PI3K. This inhibition of spreading and migration [82] could represent a mechanism for fine tuning of inflammatory cell recruitment. A similar function has been reported for soluble ALCAM, which may prevent the accumulation of premature tissue macrophages [83]. ALCAM is expressed on endothelial cells and upregulated upon inflammatory stimulation. Under homeostasis, peripheral blood monocytes and lymphocytes show only weak expression of ALCAM, whereas inflammation leads to upregulation of surface ALCAM, enabling homophilic (ALCAM-ALCAM) or heterophilic (ALCAM-CD6) interactions and supporting transmigration [83–85]. Especially on T lymphocytes, ADAM17-mediated ALCAM shedding appears to be essential for activated lymphocyte recruitment [84]. Clustering of ALCAM via upregulation of the tetraspanin CD9 was found to limit ADAM17-mediated shedding and lymphocyte recruitment [84].

Also on leukocytes themselves, ADAM10 and ADAM17 are the predominant shedding enzymes, influencing cell-cell and cell-matrix interactions. Mac-1, which is expressed on monocytes, is one of the major interaction partners for endothelial expressed RAGE. Although Mac-1 cleavage by ADAM10 and ADAM17 was shown, only spatial and temporal ADAM17-dependent cleavage is essential for transendothelial migration of monocytic cells [86]. The function of L-selectin as adhesion molecule and interaction partner of E-selectin [87] has been extensively studied in neutrophil migration. L-selectin shedding by ADAM17 limits recruitment of neutrophils at early time points during inflammation. This is mediated by weakening of adhesion during leukocyte rolling along the endothelium leading to higher velocity of rolling leukocytes. In contrast to neutrophils, monocyte recruitment is not affected by L-selectin shedding [88]. ADAM17 mediated proteolytic shedding has been reported for CXCR2, the receptor for CXCL1 and CXCL8. Stimulation by lipopolysaccharide (LPS) or formyl peptide leads to enhanced CXCR2 shedding via ADAM17 and impairs neutrophil recruitment, which can be detrimental during excessive inflammation [89]. ADAM10 and ADAM17 are both responsible for the cleavage of epidermal growth factor receptor (EGFR) ligands, resulting in transactivation of EGFR [90]. However, for leukocyte migration, a distinct ligand has not been identified so far [91]. The migration of B lymphocytes towards Notch ligands was shown to depend on ADAM17 and *γ*-secretase activity, indicating a role of Notch receptor shedding in B cell migration [92]. It is not yet clear whether this could also account for other leukocyte populations. Involvement of cleavage events in the interaction with extracellular matrix proteins has been reported for CD44, the discoidin domain receptor 1 (DDR1), and the hepatocyte growth factor (HGF) receptor c-met. CD44 interacts with hyaluronic acid and is stimulus-dependently cleaved by either ADAM10 or ADAM17, involving Ca^2+^ influx or PKC and Rac activation [93]. Proteolysis of CD44 is essential for leukocyte migration as was exemplarily shown in liver neutrophil trafficking [94]. DDR1 is expressed on neutrophils, monocytes, and lymphocytes and mediates the adhesion to collagen. DDR1-collagen interaction results in DDR1 phosphorylation and subsequent shedding by ADAM10 allowing pseudopod extension and migration through p38 and NF-kB activation [95]. c-met surface expression and signaling are regulated by ADAM10- and ADAM17-mediated shedding. Migration of G-CSF mobilized myeloid cells and hematopoietic stem cells was shown to be in parts mediated via the HGF/c-met axis, increasing matrix degradation by enhancement of MMP expression [96]. In addition, cytokines can promote the migratory response of leukocytes. For example, migration of Langerhans cells in the skin and the mucosa largely depends on IL-1 and TNF*α* [97]. Also, for IL6, a promigratory function on T cells has been described [98]. This would imply that proteolytic shedding of cytokines like TNF*α* also regulates immune cell migration, and this activity could be counterregulated by receptor shedding of TNFR, IL1R, or IL6R [97, 99, 100]. However, linking investigations are still missing.

Besides ADAM10 and ADAM17, only ADAM8 has been shown to influence leukocyte migration by catalytic activity. The P-selectin glycoprotein ligand-1 (PSGL-1) mediates leukocyte rolling on the vascular endothelium in inflammation and is further essential for the migration of T lymphocytes under homeostatic conditions. ADAM8-dependent cleavage at the uropod loosens the initial binding and facilitates leukocyte migration [101–103]. However, besides ADAM8, also ADAM10 and the aspartyl protease BACE1 have been reported to cleave PSGL-1 [104], and the relative contribution of each protease remains unclear.

### 3.2. ADAM Noncatalytic Functions in Leukocyte Migration

In addition to the proteolytic shedding, many ADAMs display noncatalytic functions that can contribute to leukocyte migration. Such activity has been reported for ADAM8, ADAM9, ADAM10, ADAM15, ADAM17, ADAM19, ADAM28, and ADAM33. Most of these functions involve the regulation of integrins, directly affecting integrin-dependent adhesion as one of the first steps in leukocyte recruitment. Adhesive contacts via integrins are required for cell motility, but too tight adhesions prevent cell migration. Regulation of integrins can be a direct result of integrin binding to noncatalytic domains of ADAMs or result from the activation of signaling pathways leading to modulation of integrin expression and activity at the cell surface.

ADAM8 is essential for the clustering of *β*_1_ integrin at the leading edge of migrating cells [48]. However, the function of this mechanism was not yet shown for leukocytic cells, but a promigratory/proasthmatic as well as antimigratory/antiasthmatic function of ADAM8 was reported in ovalbumin induced asthma [105, 106]. ADAM15 is expressed on endothelial cells and enhanced during inflammatory processes, but circulating monocytes lack ADAM15 expression [107]. ADAM15 surface expression correlates with expression of *α*_5_ integrin, thereby influencing migration through adhesion [108]. Furthermore, the cytoplasmic tail of ADAM15 induces signaling processes including ERK1/2 and Src phosphorylation, which enhance endothelial permeability and monocyte transmigration [107, 109]. ADAM28 in Jurkat cells can support *α*_4_*β*_1_, *α*_4_*β*_7_, and *α*_9_*β*_1_ dependent adhesion, whereas ADAM33 can only interact with *α*_9_*β*_1_ [110]. ADAM9 was shown to promote neutrophil activation and chemotaxis by engagement of *α*_*v*_*β*_3_ and *α*_9_*β*_1_ integrins within a hierarchical cross talk of integrins with CXCR2, involving GPCR, PI3K/AKT, and MAPK activation [111]. In myeloid cells, ADAM10 deficiency correlated with reduced surface upregulation of *α*_5_*β*_1_ integrin upon CCL-2 stimulation accompanied by impairment of p38 and Rho activation, cell-matrix interaction, and cytoskeletal rearrangement, resulting in inhibition of migration [91].

## 4. Migratory Function of ADAM Proteases in Inflammatory Diseases

The role of ADAMs in inflammatory diseases is still not fully understood. As described above, leukocytes and tissue cells express various ADAMs, which are likely to modulate cell migratory processes involved in the pathogenesis of many inflammatory diseases. As examples, we here briefly mention multiple sclerosis (MS), rheumatoid arthritis (AR), and atherosclerosis. We will here focus on the impact of ADAMs in cell migration. Other ADAM-dependent pathogenic mechanisms are reviewed elsewhere [112].

It was clearly shown that MMPs and TIMPs hold promigratory functions in MS, contributing to the pathogenesis of the disease [113]. However, ADAM proteases can be only indirectly linked to migratory processes so far. Investigation of postmortem brain sections of MS patients revealed that ADAM10 is expressed in astrocytes and sometimes in perivascular macrophages [114]. On the other hand, ADAM17 expression is associated with active MS lesions and gadolinium-enhancing lesions of relapsing-remitting MS patients [115], more specifically in blood vessel endothelial cells, activated macrophages, microglia, astrocytes [116], and invading T lymphocytes [114]. Invading T lymphocytes are one major source of TNF, which is shed by ADAM17 and associated with the relapse phase but would rather indirectly contribute to migratory processes. Further, CX3CL1, which is found in the brain and serum during MS, could fulfill different functions. On the one hand, CX3CL1 could activate and recruit circulating leukocytes, but, on the other hand, the adhesion to membrane-bound CX3CL1 could be blocked by its soluble form [117].

ADAM8, ADAM10, ADAM15, and ADAM17 have been shown to be upregulated in synovial fluid or tissue of RA patients compared to healthy individuals [47, 73, 118, 119]. ADAM17 is responsible for the strong TNF release, which orchestrates the inflammatory response in RA. However, the TNF release influences the migratory process only indirectly. Expression levels of ADAM8 and ADAM10 in the synovial fluid correlate with the degree of joint inflammation and disease severity, respectively [47, 73]. ADAM10 knockdown in RA reduced the LPS-induced migration and invasion of fibroblast-like synoviocytes (FLS) in vitro [120]. ADAM10 was clearly enhanced in biological fluids of RA patients, displaying a higher monocyte migratory activity [73]. Further, ADAM10 plays a proangiogenic role in RA indicated by an elevated tube formation of endothelial cells, which may be due CX3CL1 and vascular endothelial growth factor (VEGF) release [121]. One possible treatment of RA is tocilizumab, an antibody against the IL-6R. ADAM10 has been shown to be the major sheddase involved in induced shedding of IL-6R in human [122]. ADAM10 was shown to correlate with soluble CX3CL1, which was reduced upon antibody treatment and could serve as predictive marker [123]. However, the orchestration of the events and the contribution to cell migration of endothelial cells, FLS, and leukocytes in RA have still to be examined. Similar to ADAM10, knockdown of ADAM15 results in suppressed migration and invasion of FLS and silencing of ADAM15 in a rat model of CIA reduced the arthritis score and the extent of joint damage [124]. It was further shown that VEGF treatment enhances ADAM15 expression in synovial fibroblasts and in endothelial cells, if VEGFR2 expression was increased by TNF*α* stimulation. The overexpression of ADAM15 in RA synovium and the regulation through VEGF and VEGFR2 suggest a possible function of ADAM15 in angiogenesis in RA synovium [125].

ADAMs 8, 9, 10, 12, 15, 17, and 33 are expressed in human atherosclerotic lesions [126–128]; however, their causal role in the pathogenesis still remains ill-defined. Influences on involved migratory processes have been described only for ADAM10 and ADAM15 so far. The expression of ADAM10 in atherosclerotic lesions has been associated with human plaque progression and neovascularization [60, 127, 129]. Genetic ablation of ADAM10 or its inhibition reduces migration of endothelial cells, monocytes, macrophages, and vascular smooth muscle cells in vitro [60, 130, 131]. Further, the proatherosclerotic effect of MMP8 deficiency indicated by higher neointima formation was shown to be dependent on an ADAM10-, N-cadherin, and *β*-catenin-mediated pathway [131]. In addition, myeloid ADAM10 deficiency reduced expression of MMP9 and MMP13 as well as MMP2 gelatinase activity in macrophages. Further, ADAM10-deficient macrophages displayed a more anti-inflammatory phenotype with reduced migratory potential and less production of inflammatory mediators like TNF*α*, IL-12, and NO upon LPS stimulation. These effects together led to higher plaque collagen content, resulting in a shift from inflammation to fibrosis in atherosclerosis due to altered ECM degradation and a shift in atherosclerotic plaque stability [130]. Furthermore, ADAM15 deficiency reduced atherosclerotic lesion progression in mice due to improved endothelial barrier function and reduced monocyte transmigration via Src activation [107].

## 5. ADAM Proteases in Cell Migration during Healing Responses

Wound healing is a highly dynamic process that involves the coordinated response of a considerable number of different cell lineages in an attempt to restore tissue integrity and homeostasis and is fundamentally similar among tissue types. The wound healing process is characterized by overlapping phases of inflammation, angiogenesis, reepithelialization, and resolution [132]. These steps involve proliferative as well as migratory responses of various tissue cells and leukocytes. The role of ADAMs in leukocyte recruitment has been discussed in the previous section. For the purpose of this review, we will focus on ADAM-dependent migration in angiogenesis and reepithelialization in this section (see Table 2).

### 5.1. Angiogenesis

During wound healing, the angiogenic process is initiated, which involves sprouting of wound edge capillaries followed by invasion into the site of damage. After a few days, the microvascular network is apparent throughout the wound. Sprouting angiogenesis is a multistep process involving endothelial cell proliferation and guided migratory invasion, which are regulated by proteolytic and nonproteolytic activities of ADAMs [221].

Endothelial cells in sprouts are guided by tip cells followed by a multicellular stalk of endothelial cells, which are interconnected by VE-cadherin at cell-cell junctions and successfully form an inner lumen. One of the best studied proangiogenic signals, namely, VEGF, controls the directed migration of the tip cells [222]. ADAM10, ADAM12, and ADAM17 have been shown to shed VEGFR2 upon activation in vitro, thereby limiting vascular sprouting [60, 160, 163]. However, ADAM10 and ADAM17 can have opposing effects on physiological angiogenesis in vivo. While genetic ablation or inhibition of ADAM10 increases vascular sprouting, most likely due to reduced shedding-mediated Notch activation, ADAM17 hypomorphic mice or inhibition of ADAM17 decreases angiogenesis in vivo [154–157, 223]. Interestingly, ADAM17 may exert both positive and negative effects on angiogenesis by timely release of TNF*α* [177] or its receptor TNFR. TNF*α* stimulation increased ADAM17 expression accompanied by increased shedding of TNFR, which could be a self-protective mechanism during prolonged immunostimulation [178]. Chronic exposure to TNF*α* inhibited angiogenesis, while a 2-3-day pulse stimulated angiogenesis by inducing a migratory tip-cell phenotype [179]. The different functions of TNF*α* signaling in ischemia-mediated angiogenesis could be in part explained by the opposite effects of TNFR1 and TNFR2. Stimulation of TNFR2 enhances endothelial cell migration, whereas TNFR1 inhibits migration [180]. However, it remains elusive how and when ADAM17 acts regulatory by the shedding of TNFRs in this process. Knockdown of ADAM17 reduces the amount of invading endothelial cells as well as the distance they covered in vitro [224]. Additionally, the migration of VEGF-A- or FGF7-stimulated endothelial cells depends on EGFR activation via shedding of HB-EGF by ADAM17 [17, 172]. Migrating endothelial cells continuously extend and retract processes at the leading edge of the sprout [225]. Expression of a dominant negative mutant of ADAM17 increased the number of extending processes in endothelial cells, suggesting that ADAM17 is involved in proper retraction of these peripheral processes [224]. During angiogenesis, pericytes and smooth muscle cells are recruited to endothelial cells to stabilize developing blood vessels. This migratory process is mediated via the interaction of platelet-derived growth factor- (PDGF-) B and its receptor PDGFR*β*. PDGFR*β* is constitutively shed by ADAM10, whereas ligand-dependent activation does not lead to its own shedding but to stimulation of ADAM17-dependent EGFR ligand shedding. This PDGFR*β*-EGFR-signaling axis was shown to be critical for PDGF-B stimulated cell migration [159]. Not only PDGFR*β* but also DDR1 and its shedding may be important for angiogenesis. It was shown that DDR is critical in the regulation of attachment to collagen, chemotaxis, and MMP production in smooth muscle cells. Further, DDR1-null mice displayed lower intimal thickening after vascular injury [148]. As already mentioned before, DDR1 is shed by ADAM10 upon interaction with collagen [95]. Thus, it seems feasible that shedding of DDR1 by ADAM10 could be involved in smooth muscle cell migration and intimal thickening. However, this possibility still has to be investigated. Smooth muscle cell migration plays an important role in artery in-stent restenosis. Studies in minipigs and arterial smooth muscle cells indicated an involvement of ADAM10 in this process, partly mediated by Notch1 and Notch3 cleavage, resulting in nuclear translocation and downstream gene induction [158].

Both ADAM8 and ADAM15 are also implicated in angiogenesis. Overexpression of ADAM8 in vitro increases shedding of coexpressed proteins, including Tie2, VEGFR1 and VEGFR2, EphB2, and junctional molecules CD31 and VE-cadherin [138]. Further, ADAM8 was shown to be associated with angiogenesis after spinal cord injury in mice [139]. The expression of ADAM15 is upregulated in endothelial cells during angiogenesis, where it can interact with *α*_5_*β*_1_ and *α*_*v*_*β*_3_ via its RGD motif [167], while ADAM15 knockout mice showed reduced angiogenic responses to hypoxia [168].

### 5.2. Reepithelialization

Upon injury, the regrowth of epithelium over a damaged area is pivotal in the repair phase of wound healing. Epithelial cells at the edge of the wounded tissue are instructed to loosen their cell-cell and cell-ECM contacts and migrate across the denuded area, while epithelial cells behind the leading edge start to proliferate until a new epithelium covers the damaged tissue [132].

E-cadherin, an adhesion molecule responsible for anchoring neighboring cells to one another, is cleaved by ADAM10, thereby decreasing cell-cell contacts and increasing migration of keratinocytes [61]. Studies have reported that ADAM9, ADAM10, and ADAM15 are able to cleave ECM substrates in vitro, including fibronectin, type IV collagen, and gelatin, thereby facilitating cell migration and release of sequestered growth factors to ECM proteins [166, 226, 227]. Shedding of collagen XVII from keratinocytes by ADAM9, ADAM10, and ADAM17 reduced migration due to the inhibitory effect of soluble collagen XVII on migration [140]. In line, wound repair was accelerated in ADAM9 deficient mice due to increased migration of keratinocytes and reduced constitutive shedding of collagen XVII [141]. Additionally, binding of ADAM9 to the integrin *α*_3_*β*_1_ in keratinocytes increased MMP9 expression and subsequent cell migration [142]. EGFR ligands, including TGF-*α*, HB-EGF, amphiregulin, betacellulin, and epiregulin, are expressed by keratinocytes and are key regulators of their migration [228], which can be processed into active soluble factors by ADAM9, ADAM10, and ADAM17 [17, 90, 143, 176]. Deficiency of iRHOM2, which is critical for the maturation of ADAM17 in keratinocytes, reduces EGFR-dependent keratinocyte migration underlining the importance of ADAM17 for this response [38]. Besides the generation of soluble promigratory ligands, ADAM17 may also interfere with reepithelialization by the shedding of TNFR1 and TNFR2. Physiological levels of TNF*α* enhanced migration through TNFR2, whereas pathological levels inhibited wound closure through TNFR1 [181]. However, as already mentioned studies directly linking ADAM17, TNFR1 and migration are missing. Skin explants from ADAM12 knockout mice displayed increased migration of keratinocytes, potentially via shedding of HB-EGF and insulin-like growth factor (IGF) [162]. In contrast, wound healing in ADAM15 deficient mice was unaltered making ADAM15 dispensable for this process [229].

## 6. ADAM Proteases in Cancer Cell Migration

Tumor metastasis, the dissemination of cancer cells and subsequent outgrowth of secondary tumors at distant sites, is a major contributor to cancer morbidity and mortality. Metastasis is a multistep process involving the detachment of cancer cells from their surrounding tissue, the migration or invasion through the local extracellular matrix towards lymphatic or blood vasculature, the intravasation into the circulation, and lastly the extravasation at a distant tissue to establish a secondary tumor [230]. A number of ADAM proteins are (over)expressed in malignant tumors and have been shown to regulate several steps of the metastatic process, which will be discussed below (see Table 3).

### 6.1. Detachment and Invasion of Cancer Cells

The initial process of tumor invasion involves loss of cell-cell adhesion and increased cellular mobility, resembling many features of epithelial to mesenchymal transition program during developmental processes (e.g., loss of E-cadherin expression, increased expression of N-cadherin, and enhanced growth factor signaling) [231]. E-cadherin has been shown to be cleaved by ADAM9, ADAM10, and ADAM15 in cancer cells in vitro [61, 186, 194, 232]. The cytoplasmic domains of cadherins interact with *β*-catenin, and reduced surface expression of E-cadherin causes *β*-catenin to dissociate from the plasma membrane. When stabilized by active Wnt signaling or by mutations in the *β*-catenin phosphorylation/degradation pathway, *β*-catenin translocates to the nucleus where it modulates expression of genes involved in cell migration and invasion [233]. Additionally, soluble E-cadherin can disrupt adherens junctions and increase migration and invasion by activating EGFR signaling and upregulating several MMPs [232, 234]. ADAM10 is a major protease for N-cadherin shedding in vivo [235]. Increased N-cadherin expression enhances the migration and invasiveness of cancer cells by acquiring an affinity for mesenchymal stroma cells [231, 236]. Surprisingly, ADAM10-mediated cleavage of N-cadherin enhances the migration of glioblastoma cells in vitro [199]. Tetraspanins have been recognized as positive and negative regulators of tumor cell migration [237]. As described above, tetraspanin CD9/TSPAN28 acts as adapter molecule of ADAM17 and contributes to ADAM17 surface activity. By this, CD9 indirectly contributes to shedding of adhesion molecules such as ALCAM, which then may contribute to its reported tumor suppressive function [237]. TSPANC8 family proteins (TSPAN5, TSPAN10, TSPAN14, TSPAN15, TSPAN17, and TSPAN33) differentially regulate ADAM10 compartmentalization and thereby selectively promote cleavage of N-cadherin or CD44 which may represent critical steps in tumor cell migration [55, 238]. In addition, tetraspanin TM4SF3/TSPAN8 promotes ADAM12 upregulation and by this esophageal carcinoma cell migration [239].

Similar to keratinocytes, EGFR ligand shedding and subsequent EGFR signaling are a key factor in tumor migration and invasion [240, 241]. For instance, ADAM17 enhances migration and invasion of breast cancer and glioblastoma stem cells by promoting EGFR signaling through shedding of several EGFR ligands [214, 215, 242] and PI3K/AKT activation, for example, shown for TGF-*α* shedding in triple-negative breast cancer cell invasion [215] and EGFR activation in hypoxia-induced glioma cell invasion [243]. ADAM17 generates a transmembrane C-terminal fragment (tCTF) of syndecan-1, which is further processed by *γ*-secretase, resulting in a cytoplasmic C-terminal fragment (cCTF). Full-length syndecan-1 and the tCTF mediated lung cancer cell invasion and metastasis, whereas the cCTF antagonized these functions [174, 175].

Overexpression of ADAM8 in pancreatic ductal adenocarcinoma cells increased their migratory and invasive capacities in vitro and in vivo by enhancing ERK1/2 signaling and MMP activity and the clustering of *β*_1_ integrin [48]. Moreover, lung cancer cells express an alternatively spliced variant of ADAM8, termed Delta14, which increased lung metastasis to bone in vivo [244].

Hepatic stellate cells secrete an alternatively spliced soluble isoform of ADAM9 (ADAM9-S), which can interact with *α*_6_*β*_4_ and *α*_2_*β*_1_ integrins on colon carcinoma cells via its disintegrin domain. A similar interaction was also shown for *α*_6_*β*_1_ integrin in prostate tumor cells [187]. Integrin-bound ADAM9-S increases the invasive capacity of colon carcinoma cells by cleaving laminin and other ECM components in vitro [188]. Additionally, ADAM9-S increases breast cancer migration via its metalloproteinase activity, while the transmembrane form of ADAM9 (ADAM9-L) has the opposite effect on migration, which requires its disintegrin domain [185]. Furthermore, ADAM9 disintegrin domain mediates the interaction of fibroblasts and melanoma cells contributing to proteolytic activities required during invasion of melanoma cells [245].

ADAM10 can also generate soluble L1 cell adhesion molecule (L1CAM), which stimulates the migration of tumor cells through binding to the *α*_*v*_*β*_5_ integrin [149]. Additionally, L1CAM shedding can take place either at the cell surface or in the endosomal and exosomal compartments, which is mediated by ADAM10 [196, 246]. The resulting soluble L1CAM enhances ERK phosphorylation and cell migration [246]. In glioma cells, upregulation of ADAM10 at the tumor edges was shown to result in increased soluble L1CAM, interacting with integrin receptors, activation of focal adhesion kinases, and focal complex turnover, all resulting in enhanced migration [197]. ALCAM is also upregulated on many tumors. ALCAM shedding from epithelial ovarian cancer (EOC) cells by ADAM17 interrupts adhesive functions, which is an essential step in EOC cell migration [247]. In oral squamous cell carcinoma, overexpression of ADAM10 associates with *α*_*v*_*β*_6_ mediated invasiveness of the tumor cell [205]. In human non-small-cell lung cancer, overexpression of ADAM10 increased the migration and invasion potential of the tumor cells via the activation of the Notch 1 signaling pathway [200].

Highly metastatic tumors are associated with increased expression of ADAM12 [248]. Overexpression of ADAM12 increases migration and invasion of small cell lung cancer and head and neck squamous cell carcinoma cells [248, 249]. Further, adhesion states for migration and anchorage of melanoma cells are partly regulated by interactions of ADAM12 with *α*_9_*β*_1_ [207]. As already described for ADAM9, ADAM12 exists in a transmembrane and in a soluble form. In particular, ADAM12-S was associated with breast cancer cell invasion in vitro and metastasis in vivo [206].

ADAM15 promotes the migration and invasion of non-small-cell lung cancer cells by increasing the expression of MMP9 and the conversion of pro-MMP9 to active form [210]. In contrast, ADAM15 suppresses vitronectin- and fibronectin-induced cell adhesion and migration in ovarian cancer cells by binding to the *α*_*v*_*β*_3_ integrin via its RGD domain [212]. Interestingly, macrophage- and tumor-derived exosomes containing ADAM15 have a similar effect on cancer cell adhesion and migration via the same mechanism [250]. Similar to ADAM15, the proteolytically inactive ADAM23 can function as adhesion molecule by binding to the *α*_*v*_*β*_3_ integrin and promoting cell-cell interactions and concomitantly modulating metastasis by reducing migration and adhesion to vitronectin [220]. Hyaluronic acid, another component of the ECM, is a ligand for the cellular adhesion molecule CD44. ADAM10 and ADAM17 regulate cell-ECM adhesion by shedding CD44, thereby stimulating CD44-mediated migration of adenoma cells through Rac activation [93, 189].

### 6.2. Intra- and Extravasation of Cancer Cells

Upon reaching a blood or lymph vessel, invading cancer cells disrupt the endothelial junctions and cross the endothelium into the bloodstream. Surviving circulating cancer cells attach to endothelial cells in a similar manner as leukocytes (as discussed earlier), involving a wide range of ligands including transmembrane chemokines, selectins, integrins, cadherins, CD44, and immunoglobulin superfamily receptors, followed by transmigration.

ADAM8 stimulates endothelial transmigration of triple-negative breast cancer cells via *β*1-activation in vitro and promotes tumor metastasis in vivo [183]. Eph receptor tyrosine kinases and their membrane-bound ligands, ephrins, are implicated in a variety of cellular responses, including repulsion, attraction, and migration, depending on the cell-type and receptor-ligand binding partners [251]. Eph receptor A1 and ephrin A1 complexes maintain cell-cell adhesion between endothelial cells, however enhancing ADAM12-mediated shedding of ephrin A1 by TGF-*β*1 in primary tumors results in lung hyperpermeability that allows tumor cells to extravasate into the lungs [208]. Additionally, knockdown of ADAM15 in a prostate cancer cell line (PC-3) reduced adhesion to and transmigration through a monolayer of endothelial cells in vitro. In line, loss of ADAM15 in PC-3 cells injected in SCID mice led to an attenuated bone metastasis [209]. Cleavage of the transmembrane chemokine and adhesion molecule CXCL16 is predominantly mediated by ADAM10 [252]. Increased serum levels of shed CXCL16 have been correlated with high metastasis rate in ovarian cancer cells, which may reflect a prometastatic ADAM10 activity [191]. Moreover, soluble CXCL16 can mediate the attraction and adhesion of prostate cancer cells via *α*_*v*_*β*_3_ integrin clustering [192]. On the other hand, high expression of CXCL16 but low ADAM10 expression, which presumably leads to less CXCL16 shedding and accumulation of transmembrane CXCL16, correlated with longer survival in renal cancer patients. Interestingly, transmembrane CXCL16 in renal cancer cells was found to act proadhesive but antimigratory [193].

## 7. Conclusion and Outlook

In summary, ADAMs 8, 9, 10, 12, 15 and 17 can contribute to cell migration by interfering with distinct steps of cell migration via a number of different effector molecules. This is true for inflammatory cells, various tissue cells, and cancer cells and involves proteolytic cleavage or nonproteolytic interactions of the proteases. Importantly, to promote cell migration, these actions must become efficient at the right time and the right place. For example, shedding of adhesion molecules on leukocytes before the migration process has started will result in less adhesiveness, but once an adherent leukocyte needs to resolve adhesion in order to move to its destination shedding of adhesion molecules may be required. Furthermore, when a tumor cell acquires a motile phenotype, ADAMs can resolve adhesion contacts to the ECM, thereby allowing dissemination of the tumor, but, on the other hand, when it comes to settlement of this motile cell to form a metastasis shedding of adhesion molecules can also prevent its attachment.

Studies with genetically altered mice indicate which ADAMs may hold a promigratory function in general. Deletion of ADAM10 in leukocytes reduces leukocyte recruitment in a murine model of acute lung inflammation [91]. ADAM10 deficient hepatocellular carcinoma showed less metastasis formation in vivo [203]. Transfer experiments also showed a promigratory role of ADAM8 on leukocytes (unpublished data) and on breast and pancreatic cancer cells [48, 183]. Moreover, ADAM9 deficiency in lung cancer cells reduces metastasis in the brain [253]. However, deficiency of pathophysiologically important ADAM proteases does not necessarily reduce cell migration in general. For example, deficiency of ADAM17 in leukocytes clearly reduces inflammatory mediator production and reduces the response to bacterial sepsis [254], but the absence of this protease does not lead to diminished recruitment of inflammatory cells to inflammatory sites [88, 91, 255].

Thus, inhibition approaches for selected ADAM proteases on leukocytes or cancer cells are warranted. Inhibitor development is most advanced for ADAMs 8, 10, and 17 [32, 256]. In endotoxin-induced acute lung inflammation, treatment with a combined ADAM10/17 inhibitor was found to limit leukocyte recruitment [257]. In a murine model of allergic lung inflammation, ADAM10 inhibition could yield protective effects, which may be partly due to inhibition of leukocyte migration [258]. Interrupting ErbB receptor transactivation by a combined inhibitor of ADAM10 and ADAM17 reduced non-small-lung-cancer-cell formation, subcutaneous tumor growth, and breast cancer and fibroplasia [259, 260]. Furthermore, also ADAM8 inhibition via a neutralizing antibody or a cyclic peptide inhibitor led to a reduction of allergic lung inflammation, which is associated with a reduction of inflammatory cell infiltration [134, 261]. ADAM8 inhibition could also decrease metastasis of implanted pancreatic tumor cells [48]. ADAM12 inhibitory antibodies or genetically altered tissue inhibitor of metalloproteinases 2 (TIMP2) for specifically blocking ADAM12 or recombinant ADAM12 prodomain still need to be investigated in animal models of cancer cell migration [262, 263].

The present data indicate that inhibition of selected ADAMs may be of benefit in a situation of severe acute inflammation and/or cancer. However, as summarized in several other reviews, ADAMs also contribute to regenerative processes and additionally, for some less specific metalloproteinase inhibitors, hepatotoxicity has been reported [32, 256]. Therefore, it may be advisable to particularly target ADAMs on immune cells or cancer cells to prevent their undesired migration. The development of bivalent reagents binding to these target cells on the one hand and also specifically blocking ADAM proteases on the other hand may lead to a more specific treatment strategy. This approach may be of particular interest for breast cancers that critically depend on the generation of ErbB-family growth factors via ADAM10 or ADAM17 not only in terms of cell migration and metastasis but also in terms of cell proliferation and tumor growth.

## Figures and Tables

**Table 1:** Catalytic and noncatalytic functions of leukocytic ADAM proteases in cell migration.

Protease	Domain	Cell type	Substrate or interaction partner	Process	References
ADAM7	D	Lymphocytes	*α*4*β*7, *α*9*β*1, *α*4*β*1	Adhesion	[133]
ADAM8	?	T cells, eosinophils, dendritic cells, monocytes	?	Migration	[105, 106, 134]
un	?	Leukocytes	*α*L upregulation	Migration	Unpublished
	MP	Leukocytes	PSGL-1	Adhesion, migration	[101, 103]
ADAM9	D	Neutrophils	*αvβ*3, *α*9*β*1	Migration	[111]
ADAM10	MP	Myeloid cells, hematopoietic stem cells	c-met	Migration (ECM degradation)	[96]
	MP	Neutrophils	CD44	Migration (ECM interaction)	[94]
	indirect; MP	Mast cells	Collagen IV	SCF-induced migration	[135]
	MP	Monocytes	CX3CL1	Chemotaxis	[74]
	MP	Neutrophils/monocytes/lymphocytes	DDR1	Migration (pseudopod extension)	[95]
	MP	Neutrophils/monocytes	EGFR ligands	Migration	[91]
	MP	Monocytes	Meprin A	Migration (ECM degradation)	[75, 77]
	MP	Monocytes	Meprin B	Migration (ECM degradation)	[76]
	MP	Neutrophils	RAGE	Negative regulation of adhesion/spreading/migration	[81, 82]
	MP and/or D	Leukocytes	*α*5*β*1, basement membrane interaction, cytoskeletal rearrangement	Adhesion and migration	[91, 130]
ADAM17	MP	Monocytes, lymphocytes	ALCAM	Reduced extravasation (loss of adhesion)	[83–85]
	MP	Neutrophils	CD44	Migration (ECM interaction)	[94]
	MP	Monocytes	CX3CL1	Chemotaxis	[74]
	MP	Neutrophils	CXCR2	Reduction of recruitment	[89]
	MP	B lymphocytes	Jagged-1/Delta-like 4/Notch	Migration	[92]
	MP	Neutrophils	L-selectin	Rolling, reduction of transmigration (higher velocity, reduced adhesion), resolution of transmigration	[87, 88, 136]
	MP	Monocytes	Mac-1	End of transmigration	[86]
	MP	Langerhans cells, lymphocytes	TNFR1	Migration	[97, 137]
ADAM28	D	Lymphocytes	*α*4*β*7, *α*9*β*1, *α*4*β*1	Adhesion	[133]
ADAM33	D	Lymphocytes	*α*9*β*1	Adhesion	[133]

The process indicates the function in the presence of the ADAM protease. MP: metalloproteinase domain; D: disintegrin-like domain; ?: unknown.

**Table 2:** Catalytic and noncatalytic functions of tissue ADAM proteases in cell migration.

Protease	Domain	Cell type	Substrate or interaction partner	Process	Reference
ADAM8	D, CR	Endothelial cells	PECAM-1, isolectin B4, Tie2, VEGFR1/2, EphB2, VE-cadherin	Angiogenesis	[138, 139]
ADAM9	MP	Keratinocytes	Collagen XVII, *α*3*β*1a, MMP expression	Reduced migration/wound healing	[140–142]
	MP	Epithelial cells	EGFR ligands	Migration	[143]
	MP	Vascular smooth muscle cells	HB-EGF	Migration	[144]
	D, CR	Fibroblasts	*α*3*β*1, *α*5*β*1, *α*6*β*1	Cell-cell interaction and ECM degrading capacity	[142]
ADAM10	MP	Epithelial cells	Betacellulin	Migration	[90]
	MP	Trophoblasts	c-met	Block of invasion	[145]
	MP	Glial precursor cells	CXCL16	Migration, invasion	[71]
	MP	Mesangial cells	CXCL16	Migration	[146]
	MP	Endothelial cells	CXCL16, CX3CL1	Chemotaxis, migration, transmigration	[22, 64–67, 121, 123]
	?	Fibroblast-like synoviocytes	Cytoskeletal rearrangement, VEGFA, MMP1 and MMP3 expression, CX3CL1	Migration	[73, 120]
	MP	Epithelial cells	DDR1	Collagen-adhesion, migration	[147]
	MP	Vascular smooth muscle cells	DDR1	Migration	[148]
	MP	Epithelial cells	E-cadherin	Transmigration, keratinocyte migration	[61]
	MP	Neural cells	L1CAM	Migration	[149, 150]
	MP	Endothelial cells, epithelial cells	Meprin A, meprin B	Transmigration (ECM degradation)	[75–78]
	MP	Neural progenitor cells	N-cadherin	Migration	[151]
	MP, ?	Vascular smooth muscle cells	N-cadherin, *β*-catenin	Migration	[131]
	MP	Endometriotic cells	Neuregulins	Migration	[152]
	MP	Border cells	Notch	Migration	[153]
	MP	Endothelial cells	Notch	Limitation of vascular sprouting	[154–157]
	MP	Vascular smooth muscle cells	Notch1, Notch3	Migration	[158]
	MP	Pericytes, smooth muscle cells	PDGFR*β*	Migration	[159]
	MP	Endothelial cells, epithelial cells	RAGE	Block of transmigration	[79, 80, 82]
	MP	Endothelial cells	VE-cadherin	Transmigration	[59, 60]
	MP	Endothelial cells	VEGFR2	Limitation of sprouting	[60, 160]
ADAM12	MP	Trophoblasts	?	Cell invasion placental development	[161]
	MP	Keratinocytes	HB-EGF, IGF	Migration	[162]
	MP	Endothelial cells	VEGFR2	Limitation of sprouting	[163]
ADAM13	MP	Cranial neural crest	Cadherin-11	Migration (avoidance of contact inhibition)	[164]
ADAM15	?	Intestinal epithelial cells	?	Inhibition of migration (wound healing)	[165]
	MP	Glomerular mesangial cells	Collagen IV, gelatin, fibronectin	Migration (matrix degradation)	[166]
	?	Fibroblast-like synoviocytes	VEGFA, MMP1, and MMP3 expression	Migration	[124]
	D, ?	Endothelial cells	*α*5*β*1, *αvβ*3, Src activation, ?	Angiogenesis, transmigration	[107–109, 125, 167–169]
	D	Airway smooth muscle cells	*β*1	Adhesion, migration	[170]
ADAM17	MP	Endothelial cells	ALCAM	Block of transmigration	[83–85]
ADAM17	MP	Endometriotic cells	Amphiregulin	Migration	[152]
	MP	Mesenchymal progenitors	Amphiregulin	Migration	[171]
	MP	Trophoblasts	c-met	Block of invasion	[145]
	MP	Endothelial cells	CX3CL1	Transmigration, chemotaxis	[67, 117]
	MP	Mesangial cells	CXCL16	Migration	[146]
	MP	Endothelial cells	HB-EGF	Migration	[17, 172]
	MP	Endothelial cells	JAM-A	Block of leukocyte transmigration and endothelial migration	[62, 63]
	MP	Neural progenitor cells	L1-CAM, HB-EGF	Migration	[150, 173]
	MP	Pericytes, smooth muscle cells	PDGFR*β* -EGFR crosstalk	Migration	[159]
	MP	Epithelial cells	Syndecan-1	Migration	[174, 175]
	MP	Epithelial cells (e.g., keratinocytes)	TGF*α*, amphiregulin, epigen, VEGFR	Migration	[17, 172, 176]
	MP	Endothelial cells	TNF*α*, TNFR	Time-dependent regulation of angiogenesis	[177–180]
	MP	Epithelial cells	TNFR	Migration	[181]
	MP	Endothelial cells	VE-cadherin	Transmigration	[17, 60]
	MP	Endothelial cells	VEGFR2	Angiogenesis	[182]

The process indicates the function in the presence of the ADAM protease. MP: metalloproteinase domain; D: disintegrin-like domain; CR: cysteine-rich; ?: unknown.

**Table 3:** Catalytic and noncatalytic functions of cancer cell ADAM proteases in cell migration.

Protease	Domain	Cell type	Substrate or interaction partner	Process	Reference
ADAM8	D	TNBC, pancreatic cancer	*β*1, increase of MMP activity	Invasion, metastasis	[48, 183]
ADAM8	?	Glioma cells	?	invasion	[184]
L-ADAM9	D	Breast cancer	?	Inhibition of migration	[185]
S-ADAM9	MP	Breast cancer	?	migration	[185]
ADAM9	MP	Pancreatic cancer cells, esophageal squamous cell carcinoma	E-cadherin	Invasion, metastasis	[186]
	D, CR	Melanoma cells	*α*3*β*1, *α*5*β*1, *α*6*β*1, MMP upregulation	Cell-cell interaction and ECM degrading capacity, invasion	[142]
	D	Prostate cancer	*α*6*β*1	Inhibition of migration and invasion	[187]
	D, CR	Colon carcinoma, hepatic stellate and breast cancer cells	*α*6*β*4, *α*2*β*1, *α*6*β*1	Liver metastasis	[188]
ADAM10	MP	Pituitary adenocarcinoma, glioblastoma, tumor cells in general	CD44	Cell-contact inhibition, metastasis	[93, 189, 190]
	MP	Ovarian cancer cells, prostate cancer cells	CXCL16, *αvβ*3	Migration, metastasis, attraction of cancer cells	[191–193]
	MP	Esophageal squamous cell carcinoma, breast cancer, pancreatic cancer	E-cadherin, cadherin-catenin-interaction	Cell-cell contact, invasion, migration	[194, 195]
	MP	Pituitary adenocarcinoma, glioma	L1CAM	Migration	[149, 196–198]
	MP	glioblastoma	N-cadherin, cytoskeletal rearrangement, RAGE	Migration	[82, 199]
	MP	Melanoma, gastric cancer cells, non-small-cell lung cancer	Notch1	Migration and invasion	[200–202]
		Hepatocellular carcinoma	PI3K/AKT activation	Invasion	[203]
	?	Gliobastoma sphere-forming cells	*α*5*β*1	Inhibition metastasis	[204]
	D	Oral squamous cell carcinoma	*αvβ*6, MMP regulation	Migration and invasion	[205]
ADAM12	?	Breast cancer cells	?	Invasion, metastasis	[206]
	D, CR	Melanoma cells	*α*9*β*1	Migration	[207]
	MP	Lung tumor cells	Ephrin A1	Extravasation	[208]
ADAM15	MP	Breast cancer cells	E-cadherin	Migration	[209]
	MP	Non-small lung cancer	Pro-MMP9	Migration (ECM degradation)	[210]
	MP	Breast cancer cells	HB-EGF	Migration	[211]
	D	Ovarian cancer cells	*αvβ*3	Decrease of migration	[212]
	MP	Prostate cancer cells	?	Block of extravasation and bone metastasis	[209]
ADAM17	MP	Ovarian carcinoma, thyroid tumors	ALCAM	Invasion (loss of adhesion), metastasis	[213]
	MP	Adenoma cells	CD44	Invasion (loss of adhesion)	[93]
	MP	Breast cancer, glioblastoma	EGFR ligands	Invasion	[214–217]
	MP	Nasopharyngeal carcinoma	EGFR ligands, E-cadherin	Migration, invasion	[218]
	MP	Gastric cancer cells, liver cancer	Notch1	Migration	[201]
ADAM17	MP	Colon carcinoma	PTK7	Migration	[219]
	MP	Lung cancer cells	Syndecan-1	Metastasis, invasion	[174, 175]
	?	Gliobastoma sphere-forming cells	*α*5*β*1	Inhibition metastasis	[204]
ADAM19	MP	Retinoblastoma cells, non-small-cell lung cancer, glioma	?	Invasion, metastasis	[184]
ADAM23	MP	Breast cancer	*αvβ*3	Metastasis	[220]

The process indicates the function in the presence of the ADAM protease. MP: metalloproteinase domain; D: disintegrin-like domain; CR. cysteine-rich; ?: unknown.
